# Development and validation of an early screening tool for young children with autism spectrum disorders: the checklist for Chinese toddlers with autism

**DOI:** 10.3389/fpsyt.2026.1810421

**Published:** 2026-08-07

**Authors:** Zhi Shao, Lin Zhang, Yaru Zhang, Lei Guo, Yan Luo, Zhaoxue Meng, Guifang Kuang, Qi Hong, Xianyu Tian, Zhaohui Wang, Qin Zhao, Jianming Tan

**Affiliations:** 1 Medical Research Center for Children with Autism in Chongqing, The Ninth People’s Hospital of Chongqing, Chongqing, China; 2 Faculty of Psychology, Southwest University, Chongqing, China; 3 Department of Children Health Care, Guiyang Maternal and Children Health Care Hospital, Guiyang, Guizhou, China; 4 Department of Children Health Care, Tongzhou Maternal and Child Health Hospital of Beijing, Beijing, China; 5 Department of Mental Health, Qingdao Women and Children’s Hospital, Shandong, China; 6 Department of Children Health Care, Shenzhen Baoan Women’s and Children’s Hospital, Shenzhen, China; 7 Children Health Care Center, Xi’an People’s Hospital, Shanxi, China; 8 Rehabilitation and Treatment Center for Children with Autism in Chongqing, The Ninth People’s Hospital of Chongqing, Chongqing, China; 9 Department of Children Health Care, Leshan Maternal and Children Healthcare Hospital, Sichuan, China

**Keywords:** autism spectrum disorder, behavioral assessment, early screening, rating checklist, toddlers

## Abstract

This study aimed to develop and validate the Checklist for Chinese Toddlers with Autism, a culturally adapted early screening tool for autism spectrum disorder in children aged 12–24 months. The scale was developed by synthesizing early ASD symptom characteristics, reviewing international screening instruments, incorporating interviews with 15 medical professionals, and consulting eight experts in developmental behavioral pediatrics, child psychology, and psychometrics. The final scale comprises three dimensions (reciprocal social behaviors, social communication, interest behaviors and sensory perception) and 20 items assessed via behavioral observation. Initial validation involved 809 children (aged 12–24 months) to evaluate reliability and validity of the scale, and then determine a cutoff point to differentiate the severity levels of ASD. Further, the scale was applied in 4786 children from the neighborhood child health care clinics to test the screening value of the checklist. The results indicated that a cutoff score of 12 yielded optimal performance, with high sensitivity (0.95) and specificity (0.82). Community-based screening confirmed its efficacy in distinguishing between high likelihood and low likelihood children for ASD. Preliminary evidence indicates that the Checklist for Chinese Toddlers with Autism exhibits sound psychometric properties and may serve as an early screening tool for Chinese children with ASD aged 12–24 months, however, external validation is still required to establish its generalizability.

## 1 Introduction

Autism spectrum disorder (ASD) is a lifelong neurodevelopmental condition characterized by persistent deficits in social communication and interaction, alongside restricted, repetitive patterns of behavior, interests, or activities (1). Since Kanner’s initial description in 1943 (2), ASD has garnered significant global attention. Recent epidemiological data from China indicate a prevalence of approximately 1.8% among children aged 0–6 years (3). However, delays in diagnosis remain a critical issue. A 2024 report highlighted that it often takes over a year from initial parental concern to a formal ASD diagnosis, with only 59.02% of parents recognizing early signs before their child reaches three years of age (4).

Early identification and specific support are paramount for improving long-term outcome in ASD (5, 6). Initiating support at a younger age is strongly associated with better rehabilitation results and prognosis (7, 8). Early interventions can facilitate the acquisition of social rules and skills, reduce the incidence of challenging behaviors such as aggression and self-injury (9), enhance daily living adaptation (9, 10). Conversely, the effectiveness of rehabilitation typically diminishes with age (11, 12), and without timely access to specialized services, children may face poorer prognoses or require lifelong care (13). Identifying ASD in infancy presents challenges. Symptom onset and presentation are variable, and early signs may be subtle or not readily identified through parent reports alone (14). As the etiology of ASD remains unclear and reliable biological markers are absent (15), diagnosis continues to rely primarily on behavioral assessment (16, 17), underscoring the need for effective early screening tools.

Several instruments are commonly used for the early screening of ASD, as evidenced in the literature (17–19). These include, among others, the First Year Inventory (FYI) (20), which assesses developmental level in one-year-old children; the Early Screening for Autistic Traits Questionnaire (ESAT) (21), designed for routine screening at 14 months of age; the Modified Checklist for Autism in Toddlers(M-CHAT) (22), an early screening scale for ASD applied to toddlers aged 18–30 months; the Modified Checklist for Autism in Toddlers, Revised with Follow-up (M-CHAT-R/F) (23), which administered at 16–30 months; the Checklist for Autism in Toddlers-23 (CHAT-23) (24), a revised Chinese version of M-CHAT for children aged 18–24 months; the Autism Behavior Checklist (ABC) (25), an ASD screening checklist for children over 18 months of age and the Pervasive Developmental Disorders Screening Test-Second Edition(PDDST-II) (26), a questionnaire completed by caregivers for children aged 12–48 months. Details are shown in **Table 1**.

**Table 1:** Commonly used early screening tools for ASD.

Scale	Age (months)	Administrator	Items	Duration	Sensitivity	Specificity	References
FYI	12	Parents	63	>15min	––	––	Reznick et al. (20)
ESAT	14-15	Parents; professionals	4 14	5-10min; ––	––	––	Dietz et al. (21)
M-CHAT	18-30	Parents	23	5-10min	0.85	0.93	Robins et al. (22)
M-CHAT-R/F	16-30	Parents	20	5-15min	0.85	0.98	Robins et al. (23)
CHAT-23	18-24	Parents	23	5-10min	0.84-0.93	0.77-0.85	Wong et al. (24)
ABC	>18	professionals	57	10-20min	0.38~0.5	0.76~0.97	Krug et al. (25)
PDDST-II	18-48	Parents	22	10-20min	0.92	0.91	Siegel (26)

However, many of these tools present significant limitations. Predominantly designed as parent-report questionnaires, they are vulnerable to biases arising from caregivers’ varying levels of knowledge and subjective interpretations (27, 28). In contrast, direct behavioral observation by trained professionals can provide a more objective assessment of subtle social-communication deficits (17). Furthermore, while behavioral markers often emerge between 12–24 months—and sometimes earlier (29)—many existing screening instruments are designed for children over 18 months of age (6). This creates a notable gap in the ability to screen at younger ages (30). Developing tools based on professional behavioral observation for children as young as 12 months is crucial for capitalizing on early brain plasticity and initiating services promptly.

In China, most screening and diagnostic tools are adapted from Western instruments. Although localized versions like the CHAT-23 (24) and a modified M-CHAT (22) exist, their validation often relies on small-sample studies, and they have not seen widespread clinical implementation. The scales recommended by China’s 2010 guidelines (31)(ABC and Clancy Autism Behavior Scale) demonstrate only low to moderate sensitivity and specificity, potentially leading to misdiagnosis (32, 33). Furthermore, differences in language characteristics and cultural norms—such as children’s language development, social behaviors, and parenting beliefs—may limit the applicability of imported screening scales to local populations (18, 34, 35). Studies have reported higher false-positive rates of the M-CHAT among ethnic minorities (34, 36), and the validity of the M-CHAT-R/F has been questioned when used with non-Caucasian, female, or non-urban populations (37). Therefore, there is a clear need for a culturally adapted, cost-effective, and easily administered screening tool grounded in China’s sociocultural context and the specific behavioral phenotypes of Chinese children with ASD.

Research has advanced the understanding of early ASD behavioral markers. These include diminished visual attention to social stimuli, particularly reduced eye contact (38); deficits in joint attention emerging as early as 14–15 months (39, 40); and impairments in verbal and non-verbal communication (41). Additionally, restricted/repetitive behaviors and sensory abnormalities are often present (42–44). Empirical evidence indicates that infants later diagnosed with ASD exhibit early behavioral atypicalities by 12 months of age, including diminished social communication capacities—such as reduced directed gaze, diminished vocalizations directed toward others, and impaired joint attention—as well as heightened repetitive interests and stereotyped motor behaviors (45, 46). Furthermore, prospective studies of high likelihood infant siblings have identified additional prodromal markers prior to 12 months, notably attenuated motor control (47, 48) and atypical patterns of visual attention to both social and nonsocial stimuli (49). These core behavioral domains align with DSM-5 diagnostic criteria and form the basis of many established screening tools. For instance, discriminative items in the M-CHAT relate to joint attention and social communication (22), while the STAT assesses play, requesting, and imitation (17). The CHAT-23 behavioral observation components also target similar social-communication and behavioral domains (24, 50).

In summary, this study integrated established international screening scales, DSM-5 diagnostic criteria, and previous research on early ASD behavioral markers to develop the Checklist for Chinese Toddlers with Autism (CHCTA). This novel tool employs a structured behavioral observation approach across three core domains: reciprocal social behaviors, social communication, interest behaviors and sensory perception. It is specifically designed for children aged 12 to 24 months. The objectives of this research were to evaluate the psychometric properties and clinical utility of the CHCTA within community settings, with the ultimate aim of facilitating earlier identification of ASD risk.

## 2 Study 1: development of the CHCTA and determination of cutoff scores

### 2.1 Participants

A two-stage cross-sectional design was employed. Initially, 909 clinical cases aged 12–24 months were recruited from child healthcare or psychiatric clinics in tertiary hospitals across seven Chinese cities (Beijing, Shenzhen, Chongqing, Qingdao, Xi’an, Leshan, Guiyang). These cases were used to develop the CHCTA scale, evaluate its psychometric properties—including internal consistency, test–retest reliability, and construct validity—and establish its optimal cut-off value. Participants underwent diagnostic assessments including the Gesell Developmental Schedules (Gesell) (51). Follow-up evaluations after age 2 included the Childhood Autism Rating Scale (CARS) (52) and Psychoeducational Profile-Third Edition (PEP-3) (53).

Administration and scoring were conducted by trained healthcare professionals in clinical settings (in China, pediatricians in child healthcare or child development clinics at regional tertiary hospitals bear the principal responsibility for neurodevelopmental monitoring in children (54)). The assessors were licensed healthcare professionals—specifically physicians or registered nurses—with a minimum of five years of clinical experience in ASD screening and diagnostic evaluation. Prior to data collection, subject-matter experts delivered standardized training to the assessors, covering scale administration protocols, item-level scoring criteria, and hands-on practice sessions. The assessment results are uniformly managed by an expert team. For high likelihood cases marked by scale screening or children whose parents have concerns, further consultations and follow-ups are conducted by experts.

Group assignment was aligned with the core psychometric properties of the tool. The ASD group evaluated sensitivity—the capacity to correctly identify true cases and minimize missed diagnoses, which is fundamental to screening effectiveness. The TD group assessed specificity, controlling for false positives and excluding unaffected individuals. Critically, a DD group was included to test discriminant validity: because ASD and non-autistic developmental delay exhibit marked overlap in language and social symptoms, comparison with TD alone would inflate specificity and fail to capture the tool’s ability to differentiate clinically similar conditions. At each sampling site, clinical diagnoses of childhood ASD and non-spectrum disorders were made by experienced, licensed clinicians (with vice senior title or above) based on a comprehensive evaluation of information and clinical observations in accordance with the DSM-5 diagnostic criteria (55). All children with ASD were diagnosed or excluded through the Gesell (51), CARS (52), and PEP-3 assessments (53); children with non-spectrum Developmental Delay (DD) were excluded from ASD based on their CARS scores. For Gesell, at least 2 of the developmental quotient (DQ) scores in adaptive behavior, gross motor or fine motor skills, language, and personal-social domains were <75, and they met the diagnostic criteria for DD in the DSM-5. Typical Development (TD) children were excluded from ASD and DD through PEP-3, CARS, and Gesell, and throughout the study, there was no suspicion that they had any related developmental disorders.

### 2.2 Procedure

#### 2.2.1 Scale development

##### 2.2.1.1 Dimension determination

Preliminary dimensions were derived from a systematic review of established ASD scales and the DSM-5 diagnostic criteria. Second, an open-ended questionnaire was administered to 15 autism diagnosis and assessment experts (7 pediatricians, 2 psychological and behavioral assessors, 3 clinical psychologists, and 3 special education teachers) to evaluate the proposed dimensions and supplement early ASD signs before 24 months of age, such as eye contact, response to stimuli, and social smiling. Third, in combination with the descriptions of each dimension, opinions were solicited and discussed with 6 experts in the fields of developmental-behavioral pediatrics (2 experts), autism rehabilitation (1 developmental psychologist and 1 special education expert), and psychometrics (2 experts). Finally, 3 assessment dimensions were determined, namely: reciprocal social behaviors, social communication, interest behaviors and sensory perception.

##### 2.2.1.2 Item development

Items were systematically developed based on an analysis of the core components corresponding to each dimension. The process drew upon relevant literature, existing assessment scales, and clinical experience. As a behavioral observation-based screening tool, each item of the CHCTA primarily describes target behaviors that represent the core features of its respective dimension. Key sources for item development included: adapting items from established domestic and international ASD screening tools; incorporating expert feedback and suggestions collected through the open-ended questionnaire; and integrating insights from clinical diagnostic practice.

##### 2.2.1.3 Finalization

In order to ensure the content validity of the project pool, this study also invited 8 experts from the fields of developmental behavioral pediatrics (including 2 chief physicians and 1 university professor), child developmental psychology (including 1 chief physician and 2 university professors), and psychometrics (2 university professors). These experts systematically assessed the conceptual validity of each project description, the representativeness of the behavioral exemplars, the theoretical alignment between each item and its corresponding dimensions, and the cultural appropriateness of the instrument for the Chinese context. Following several rounds of iterative revision informed by expert feedback, the final version of the CHCTA, comprising 20 items was established. The core components of reciprocal social behaviors encompass responsive attention (39, 40), imitation ability, and play skill—constructs empirically supported in the literature (17); social communication encompasses eye contact (56, 57), verbal expression, nonverbal communication (41), point towards the target and use of nonverbal actions (58); the domain of interest behaviors and sensory perception covers body postures, restricted interests and repetitive behaviors (42, 44), auditory responses, sensory responses (43), and transfer competence. A detailed description of the content corresponding to each scale item is provided in **Table 2**.

**Table 2:** Items and descriptions of the CHCTA scale.

Dimensions	No.	Items	Item descriptions
Reciprocal social behaviors	P2	Name response	When the assessor calls the child’s name, observe if the child can turn his or her head to look at the assessor
	P4	Initiating joint attention	Observe whether the child tries to use one or more ways, such as eyes, movements, sounds, etc., to draw the attention of the caregiver or the tester to the item
	P6	Play skills	To see if children could play with a toy with at least three plays in two or more ways
	P7	Social smile	When the caregiver or evaluator uses a smile to interact with the child, observe whether the child can respond with a smile or in an appropriate manner
	P8	Pretend play	Ask the child to pretend to play an item as another one.
	P10	Responding to joint attention	Observe child’s eye tracking following rater’s pointing
	P12	Imitation ability	Observe child’s imitating ability of other’s behavior
	P13	Compliance with instructions	Observe children’s ability to respond to familiar single-step oral instructions
	P18	Initiative communication	Observer child’s communicating and interacting behavior through the test
	P19	Separation anxiety	When the caregiver leaves the child, observe whether the child shows any inappropriate behavioral reactions such as restlessness, anxiety or distress.
Social communication	P5	Eye contact	Raters play hide and seek with the child, observe whether the child can look into the assessor’s face
	P11	Point towards the target	Children were observed whether they could use their fingers to point at objects during the test
	P14	Verbal expression	Observe whether children can use simple words to express requirements during the test
	P15	Non-verbal communication	Observe whether the child can understand the posture of the caregiver
	P16	Use of nonverbal behaviors	Observe and document children’s nonverbal communication behaviors during the assessment, including posture and gesture use—for instance, hand-waving to signal farewell or open-armed gestures to convey affection or receptivity.
Interest behaviors and sensory perception	P1	Comfort of physical contact	When the foster carer holds the child, observe whether the child can snuggle comfortably on the chest of the foster carer
	P3	Restricted interests and repetitive behaviors	The rater arranged the blocks into a neat row, shuffled them after completion, and observed the children’s preference for regular arrangement of the block toys
	P9	Auditory responsiveness	Observe children’s behavioral responses to a series of daily sounds, such as telephone ringing, background human chatter, car sounds, and coughing, were observed
	P17	Transfer competence	Observe children’s behavioral responses to activity transitions during the test process
	P20	Perceptual response	Observe the children’s responses to tactile and other sensory perceptions during the test process

##### 2.2.1.4 Administration and coding of behaviors

The CHCTA is administered in a professional assessment room, where target behaviors are evaluated through a battery of standardized interactive activities utilizing a designated assessment kit (including a set of building blocks, a mechanical toy, a functional toy, rectangular yellow plastic foam, a telephone-shaped toy car, a sound recorder with various sounds, and a track with a small vehicle). The trained professional engages with the child and employs systematically structured procedures to elicit the following specific target behaviors: restricted interests and repetitive behaviors, initiating of joint attention, eye contact, play skills, pretend play, imitation ability, and auditory responsiveness. Operational definitions for the elicitation of these behaviors are as follows: restricted interests and repetitive behaviors are assessed by observing the child’s reaction of distress or anxiety when the assessor deliberately disrupts or overturns neatly arranged blocks. Initiating joint attention is evaluated by activating the mechanical toy and observing whether the child actively directs the assessor’s attention toward the moving object. Eye contact is assessed by monitoring the child’s gaze toward the assessor’s face during a peekaboo interaction, a context expected to naturally engage the child’s social attention. For play skills, observed the child’s manipulation of the telephone-shaped toy car to determine the capacity for multifunctional object operation. In the case of pretend play, the examiner modeled telephonic behavior by holding the yellow rectangular plastic foam block to their ear as if using it as a telephone, then handed the object to the child and observed whether the child spontaneously engaged in complementary pretend-phone behavior. For imitation ability, after the child attends to the assessor, the instruction “do as I do” is given, accompanied by the action of clapping the assessment box with both hands; the child’s accuracy in imitating the assessor’s action is observed. Auditory responsiveness is assessed by playing a series of daily sounds (e.g., telephone ringing, background human chatter, car sounds, and coughing) to determine whether the child exhibits aversive reactions.

Each procedure is performed a preset number of times; if the infant is distracted or inattentive to the current task, the trial is repeated up to a maximum of five times. The entire CHCTA assessment takes approximately 15–20 minutes to complete. The assessment typically commences with an observation of comfort of physical contact as the primary caregiver enters the evaluation room with the child, but the order of tasks can be flexibly adjusted according to the infant’s interests. Items such as response to name, social smiling, verbal expression, use of nonverbal behaviors and compliance with instructions are interspersed among other structured activities and engagement, with two free-play sessions also included—during which the assessor places tools in front of the infant to allow for uninhibited exploration. These activities are designed to optimize the infant’s comfort level and maximize opportunities for spontaneous social vocalization. Observations of the remaining target behaviors (refer to **Table 2**) are conducted continuously across the interactive play assessment.

All target behaviors are operationally defined and scored on a 0–2 scale: 0 indicates the presence of the target ability or the absence of the problematic behavior; 1 indicates partial presence of the ability or the mild manifestation of the problematic behavior; 2 indicates complete absence of the ability or severe occurrence of the problem behavior. For the imitation ability item specifically, a score of 0 indicates successful bimanual imitation; 1 indicates the child shows imitation intent but with diminished accuracy (e.g., unimanual imitation); 2 indicates no observable imitative attempt. For the auditory responsiveness item, 0 indicates no aberrant reaction to sound; 1 indicates mild distress or anxiety toward auditory stimuli; 2 indicates severe discomfort and significant anxiety. Scoring criteria for all other items are uniform across the 12- to 24-month age range, with evaluation focused on the quality and consistency of behaviors, which are assigned and coded by the assessor based on observation. The total scale score is derived from the sum of items 1 through 20, yielding a potential range of 0–40, with higher cumulative scores indicative of elevated ASD risk.

#### 2.2.2 Reliability and validity evaluation

##### 2.2.2.1 Reliability

Eighty children were selected from clinical cases using random sampling to assess test-retest reliability, including 22 with ASD 16 with DD, and 42 with TD. All participants were reassessed two weeks after the initial evaluation. Additionally, a separate random sample of 102 children (47 ASD, 19 DD, 36 TD) was drawn from clinical cases for inter-rater reliability testing. Two standardized trained assessors, blinded to the children’s diagnoses and not involved in other developmental assessments, independently administered the test within a 30-minute interval under comparable testing conditions. Subsequently, the intraclass correlation coefficient (ICC) was used to assess inter-rater reliability. Based on the study design, a two-way random-effects model was selected, with absolute agreement as the type of measurement, and statistical analysis was conducted using the mean scores from the two raters. Internal consistency reliability was evaluated using Cronbach’s α.

##### 2.2.2.2 Validity

Content validity was evaluated by eight experts using a 3-point scale, from which the scale-level content validity index (S-CVI) was calculated. Meanwhile, two groups of age-matched analysis samples were randomly divided from clinical samples for structural validity test: 300 cases (including 85 ASD cases and 115 non-ASD cases) were used for exploratory factor analysis; Another set of 461 samples (including 126 ASD cases and 335 non-ASD cases) underwent confirmatory factor analysis using Mplus 8.3 software. Both discriminant and convergent validity were assessed. According to the parameter estimation requirements for CFA (i.e., 10 cases per item), the sample size far exceeded the minimum requirement. Criterion-related validity was determined by calculating Cohen’s kappa coefficient between scores on the CHCTA and those on the CARS.

##### 2.2.2.3 Determination of the optimal cutoff score

A total of 148 children—drawn from clinical cases and independent of the scale development process—were recruited to establish the cut-off score for the CHCTA scale (see **Table 3**). Participants were first stratified by clinical diagnosis and sequentially assigned unique identification numbers; subsequently, they were randomly allocated into two independent subsamples using a computer-generated random number sequence. The CHCTA scale was then administered separately to each subsample. In the development sample, the diagnostic performance of CHCTA for ASD was assessed via receiver operating characteristic (ROC) curve analysis. The optimal diagnostic threshold was determined by maximizing the Youden index (defined as sensitivity + specificity-1), and the corresponding sensitivity, specificity, and Youden index were reported. The 95% confidence interval (CI) for the AUC was estimated using the Bootstrap method with 1000 resamples. The validity of this cutoff was subsequently tested in the independent validation sample by calculating its sensitivity, specificity, Positive Predictive Value (PPV), and Negative Predictive Value (NPV).

**Table 3:** Age distribution of the clinical sample.

Age (months)	Development sample (n=74)			Validation sample (n=74)		
	ASD	DD	TD	ASD	DD	TD
12-14	4	4	24	4	3	22
15-17	5	0	0	8	3	1
18-24	15	21	1	12	19	2
Total	24	25	25	24	25	25

### 2.3 Auxiliary diagnostic tools

1. Gesell development schedules (51); a psychometric tool used to evaluate children’s developmental level from 0–6 years.
2. Childhood Autism Rating Scale (CARS) (52); an observational tool for evaluation of autistic children’s speech, behavior, and sensory perception.
3. Psychoeducational Profile-Third Edition (PEP-3) (53); an individualized assessment evaluating children’s developmental status and deviations from typical development for children with autism and other developmental disorders.

Additionally, a semi-structured interview based on the DSM-5 diagnostic criteria for ASD was administered, and the medical histories of the children and their parents or relatives were recorded during the interview.

### 2.4 Results

#### 2.4.1 Reliability

The test-retest reliability of the CHCTA score was 0.85 (p < 0.001), indicating high reliability. The ICC was 0.76 (95% CI: 0.61–0.85, p < 0.001), indicating good inter-rater reliability. The scale demonstrated good overall internal consistency, with a Cronbach’s α coefficient of 0.85.

#### 2.4.2 Validity

**Figure 1:**
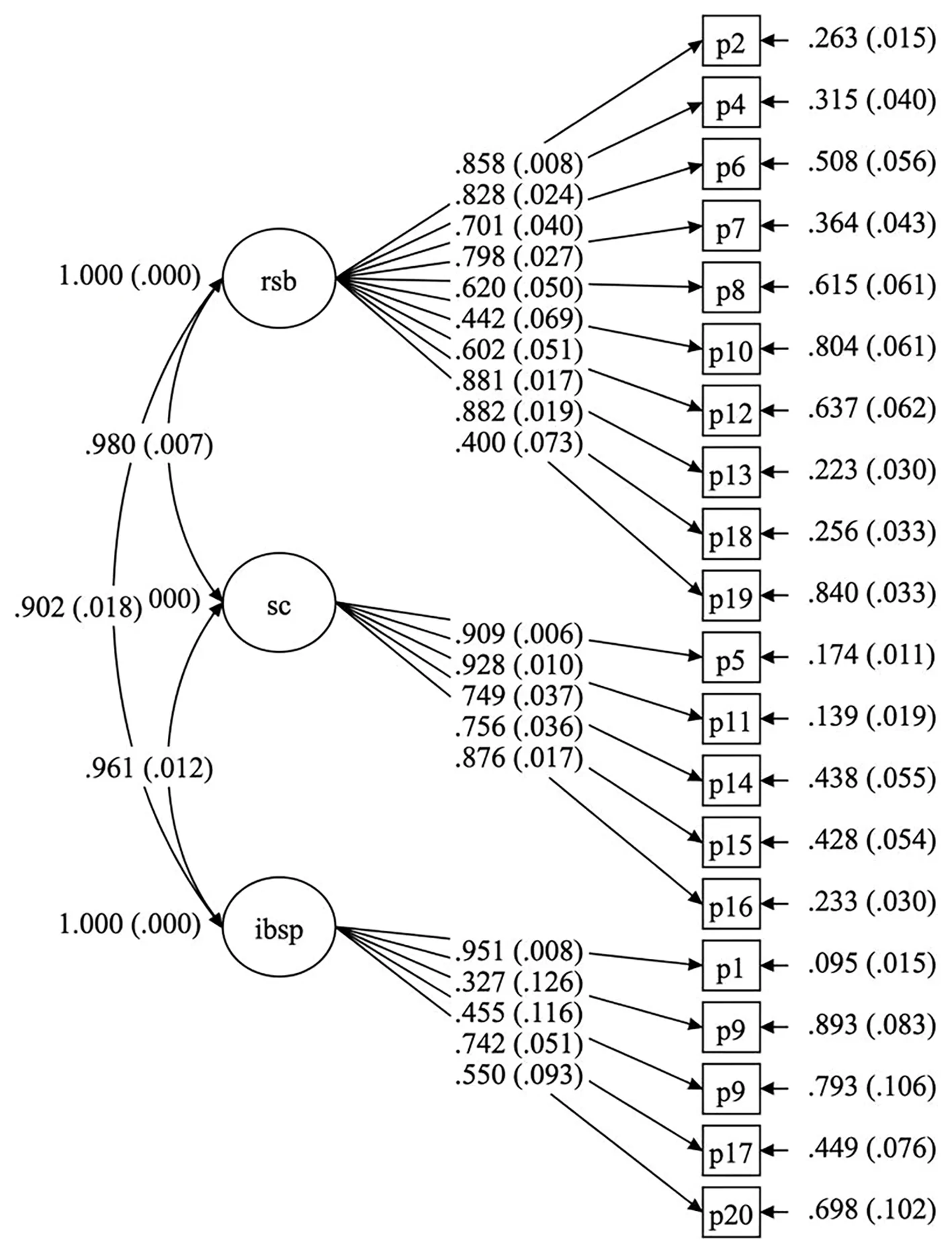
The CFA model diagram of the CHCTA. RSB, reciprocal social behaviors; SC, social communication; IBSP, interest behaviors and sensory perception.

The S-CVI was 0.94, indicating that the instrument demonstrates strong alignment with the intended construct and possesses good content validity. EFA indicated that the data were suitable for factor analysis (KMO = 0.84, Bartlett’s test *p* <.001). CFA was subsequently conducted, and model fit indices (see **Table 4**) supported the hypothesized three-factor structure over a single-factor model, confirming the instrument’s robust structural validity (59). Specific fit indices were as follows: χ²/df=2.150, CFI = 0.912, TLI = 0.904, RMSEA = 0.078, SRMR = 0.048. The standardized factor loading of each item ranges from 0.40 to 0.90, and only the standardized factor loading of item 3 is 0.327, as shown in **Figure 1**. Furthermore, discriminant validity was supported (see **Table 5**), and convergent validity was satisfactory, with all dimensions demonstrating an AVE greater than.40 and a CR above.60 (see **Table 6**). Finally, criterion validity was found to be strong, as evidenced by a Cohen’s kappa of 0.870 between the CHCTA and the CARS.

**Table 4:** Model fit indices for the CFA of the CHCTA.

Models	$\chi^{2}$	*df*	$\chi^{2}$ */df*	*CFI*	*TLI*	*RMSEA*	*SRMR*
20-3	408.531	190	2.150	.912	. 904	.078	. 048
20-1	886.518	170	5.215	.718	.685	.096	.067

**Table 5:** Discriminant validity of the CHCTA.

Dimensions	RSB	SC	IBSP
RSB	–		
SC	.228***	–	
IBSP	.047***	.055***	–
$\sqrt{AVE}$	.680	.716	.645

*** p<.001.

**Table 6:** AVEs and CRs of the CHCTA.

Dimensions	AVE	CR
RSB	.563	.781
SC	.512	.752
IBSP	.416	.653

#### 2.4.3 Cutoff score analysis

**Figure 2:**
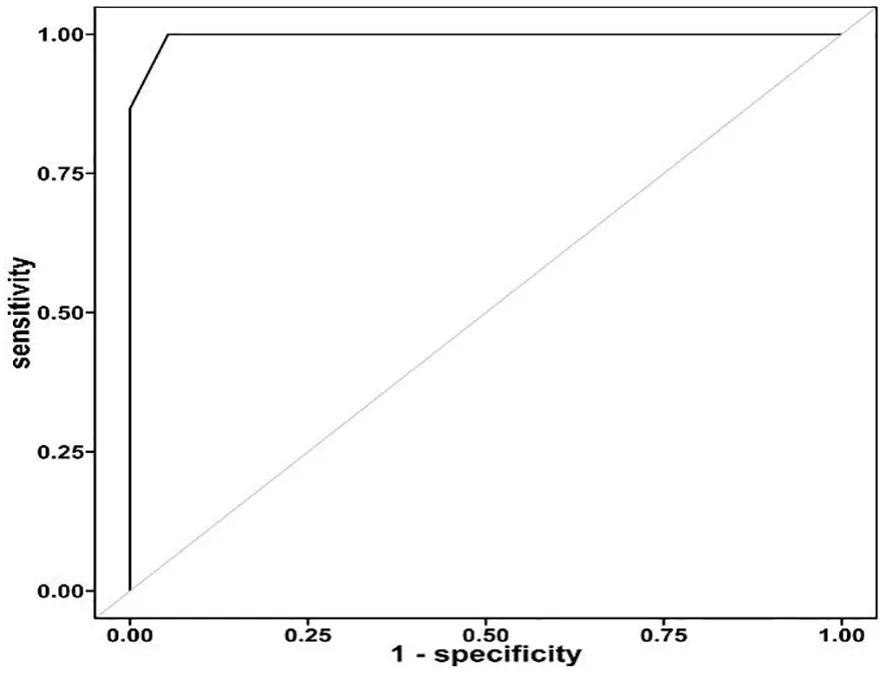
ROC curve for the best cut-off value of CHCTA.

The ROC curve analysis based on the binary classification framework of ASD and non-ASD subjects showed that the area under the curve (AUC) was 0.996 (95% CI: 0.991-1.000; **Figure 2**).

Based on the principle of maximizing the Youden index (Youden index = 0.75), the optimal cutoff value for the scale was determined to be 12 points, corresponding to a sensitivity of 0.95 and a specificity of 0.80 (see **Table 7**), indicating that this cutoff value provides a good balance between true positive and false positive rates. Validation in an independent sample confirmed the robustness of this cutoff, with a sensitivity of 0.95, specificity of 0.82, PPV value of 0.73, and NPV of 0.95. Accordingly, a total score of 12 or higher was established as the cutoff indicative of a high risk for ASD. Therefore, the preliminary evidence derived from the independent samples indicates that a cutoff value of 12 is reasonable.

**Table 7:** The cutoff score indexed by Youden index.

Cutoff score	Sensitivity	Specificity	Youden’s index
3	1	0	0
4	1	0.007	0.007
5	1	0.013	0.013
6	1	0.046	0.046
7	1	0.105	0.105
8	1	0.203	0.203
9	1	0.392	0.392
10	1	0.556	0.556
11	0.96	0.600	0.56
12	0.95	0.800	0.75^a^
13	0.91	0.812	0.720
14	0.88	0.821	0.70
15	0.78	0.948	0.728
16	0.741	0.967	0.708
17	0.616	0.982	0.596
18	0.50	0.981	0.480
19	0.446	0.98	0.426
20	0.411	0.993	0.404
21	0.321	0.993	0.314
22	0.223	1	0.223
23	0.179	1	0.179
24	0.143	1	0.143
25	0.116	1	0.116
26	0.045	1	0.045
27	0.027	1	0.027
29	0	1	0

The maximal Youden index was found when the cutoff score was 12.

## 3 Study 2: clinical application of the CHCTA

### 3.1 Participants

The CHCTA was administered in community child health clinics across the same seven cities, yielding 4786 valid screenings (3221 males, mean age = 14.59 ± 2.51 months; 1565 females, mean age = 14.87 ± 2.51 months). This sample size was considered deemed adequate, as exceeded the number of the minimum sample required of 190 cases suggested by Soper (60) to detect the size of the average effect (r = 0.3 in Cohen classification, (61)) as significant to 5%, with a statistical power of 0.8.

### 3.2 Procedure

The CHCTA was administered by trained local healthcare workers. Children with scores ≥12 were classified as high likelihood (screen-positive). All high likelihood children, those with significant concerns raised by either physicians or parents, and a randomly selected 5% sample of low likelihood (screen-negative) children were followed longitudinally. They received a final diagnostic assessment around 24 months of age by experienced clinicians based on the relevant developmental scales (such as PEP-3; CARS; Gesell) and clinical observations according to the DSM-5 diagnostic criteria.

### 3.3 Results

Among the 4786 children screened, 107 (2.24%) were identified as positive. Follow-up diagnostic outcomes for these children were presented in **Table 8**. Of the 107 screen-positive cases, 76 were subsequently diagnosed with ASD, 29 with DD, and 2 with TD. Among the 234 randomly followed low likelihood (screen-negative, score>12) children, only one child received an ASD diagnosis. The distribution characteristics of screening scores are detailed in **Table 9**. In this community-based sample, the CHCTA demonstrated excellent screening performance: sensitivity =0.99 (95%CI, 0.93-0.98), specificity =0.88 (95%CI, 0.84-0.92), PPV = 0.71 (95%CI, 0.62-0.78), and NPV = 0.99 (95%CI, 0.98-1.00).

**Table 8:** Characteristics of the clinical application sample.

N (M/F)	ASD (61/16)_M ± SD_			DD (24/5) _M ± SD_			TD (165/70) _M ± SD_		
Age (months)	12-14	15-17	18-24	12-14	15-17	18-24	12-14	15-17	18-24
Positive	12_19.50 ± 4.25_	21_20.05 ± 2.86_	43_21.09 ± 3.65_	0	8_16.25 ± 0.96_	21_15.93 ± 2.81_	0	1_14_	1_14_
Negative (5%)	1_7_	0	0	0	0	0	145_9.41 ± 1.48_	52_9.28 ± 1.22_	36_10.07 ± 1.07_

“_M ± SD_” denotes the mean score and standard deviation obtained by children in the specified age and group on the CHCTA screening assessment.

**Table 9:** Distribution characteristics of screening scores in clinical application sample.

Screening score	Positive			Negative (5%)		
	ASD	DD	TD	ASD	DD	TD
4-8	--	--	--	1	--	55
9-12	--	--	--	--	--	178
13-14	2	15	2	--	--	--
15-16	3	8	--	--	--	--
17-18	26	5	--	--	--	--
19and above	45	1	--	--	--	--

Additional metrics further supported the robustness of the CHCTA: balanced accuracy was 0.80, the F1 score was 0.82, and Matthews Correlation Coefficient was 0.78. The results collectively indicate that the CHCTA yields reliable screening outcomes in large-scale community implementation, the rationality of its cut-off value is preliminarily verified.

## 4 General discussion

This study developed and validated the CHCTA, an early behavioral observation screening tool for ASD in Chinese toddlers aged 12–24 months. The scale demonstrated excellent psychometric properties, including good reliability, content validity, structural validity, and criterion-related validity. The ICC coefficient was 0.76. According to the guidelines by Koo and Li (62), an ICC > 0.90 is considered excellent, 0.75–0.90 is good, 0.50–0.75 is fair, and < 0.50 is poor. Therefore, the inter-rater reliability in this study can be regarded as good. Its high sensitivity (0.95-0.99) and specificity (0.82-0.88) in both clinical and community samples indicate that this detection method can effectively identify children at high likelihood of ASD in specific age groups.

Confirmatory factor analysis (CFA) results indicated that item loadings on the scale ranged from 0.40 to 0.90, with the exception of Item 3 (loading = 0.327). This pattern supports the theoretical dimensionality and item allocation of the scale, aligning with both its original construct design and empirical evidence. Overall, the scale demonstrates satisfactory structural validity (63). Notably, Item 3 mainly examines the repetitive interests or behaviors of children, which is one of the core symptoms of ASD. Previous studies have shown that early ASD children exhibit restricted and repetitive behavioral patterns and interests, mainly including intense focus on specific topics or activities, repetitive physical movements, or excessive reliance on daily routines (8, 9). Based on this evidence, the research team decided to retain this item after comprehensive consideration. ROC curve analysis identified 12 as the optimal cut-off score for the scale, yielding high sensitivity and specificity. Subsequent preliminary clinical validation confirmed these findings: the cut-off demonstrated excellent sensitivity and good specificity. The AUC of 0.996 indicates excellent ASD discriminatory performance of the CHCTA; however, potential biases from sample characteristics and possible overfitting warrant careful consideration. Specifically, First, because the ROC curve analysis was based solely on binary classification—distinguishing the heterogeneous ASD group from the non-ASD group—it may inflate the AUC estimate. This concern was corroborated by subsequent clinical screening, in which all children diagnosed with DD were classified as ASD-positive. Second, despite broad geographic coverage, the relatively modest total sample size increases the risk of overfitting. Third, reliance on the total score rather than specific items or item sets may further contribute to AUC inflation. Therefore, children screening positive should undergo comprehensive evaluation integrating other developmental assessments with clinical observation and follow-up by qualified professionals to minimize the risk of false positives.

A total of 77 children diagnosed with ASD were identified during the clinical screening process utilizing CHCTA. The male-to-female ratio was approximately 4:1, which was basically consistent with the sex ratio of ASD incidence reported in previous studies (55). Furthermore, this study revealed 32 diagnostic discrepancies: 31 cases of “false-positive” diagnoses and 1 case of “false-negative “ diagnosis. Further analysis showed that the 31 “false-positive” cases included 29 DD children and 2 TD children. These “positive” children performed poorly on items of imitation ability (mean score=2), gesture use (mean score=2), and communication behavior (mean score=2). The potential reasons might be associated with the similar behavioral patterns between young children with DD and those at high risk of ASD, that they would both manifest inabilities in imitation, use of non-verbal clues, and communication behavior (64, 65). Moreover, performance, such as imitation and communication behavior, correlates significantly to the adaption to the assessment context for TD. In view of this, before administration, children should have sufficient time to adapt to the testing environment and get familiar with the administrator, lest they do not show the social abilities they have already acquired. Among the children with negative screening results but non-TD at follow-up, one case was diagnosed with ASD. Further analysis revealed that the child was only 12 months old at the time of screening, it was possible that the core symptoms had not yet fully emerged, which led to the missed diagnosis. The above results indicate that, under the optimal cut-off value condition, the screen-positive cases are mainly concentrated in the ASD children, with a certain proportion of false-positive and false-negative cases. Among the false-positive cases, 29 children with DD were included. These results suggest that the scale demonstrates high sensitivity—effectively detecting the majority of children with ASD—but comparatively lower specificity. Given the young age of the study participants, clinicians using the CHCTA for ASD risk screening should exercise caution regarding potential false positives, particularly when interpreting borderline or near-threshold scores. Furthermore, clinical follow-up data reveal a score-dependent diagnostic pattern: children scoring between 12 and 16 exhibit a significantly higher likelihood of receiving a DD diagnosis than an ASD diagnosis; conversely, scores ≥17 are associated with a higher likelihood of being diagnosed with ASD. Consequently, in clinical practice—especially during early screening of infants and toddlers—careful attention must be paid to the risks of both misdiagnosis and missed diagnosis, with particular emphasis on cases where screening scores fall near the established cut-off. To enhance diagnostic accuracy, it is recommended that clinicians integrate complementary standardized developmental assessment tools (e.g., Gesell Developmental Schedules or Bayley Scales of Infant and Toddler Development) to confirm or rule out DD. This imposes rigorous professional demands on examiners: assessment should be conducted by practitioners with substantial experience in early childhood developmental evaluation, and results should be cross-validated and comprehensively interpreted in conjunction with other developmental diagnostic tools (e.g., ADOS-2). Moreover, when communicating results to parents, clinicians should adopt an objective and prudent approach, fully accounting for the scale’s specificity limitations and the plasticity of early childhood development, thereby avoiding overinterpretation and minimizing.

The CHCTA is an early screening tool for ASD developed for Chinese toddlers. It contains 20 items and three structural dimensions. Compared to other screening instruments, CHCTA has demonstrated several advantages. First, CHCTA can be applied in toddlers aged around 15 months, which facilitates earlier diagnosis and timely intervention for young children with ASD. Moreover, researchers point out that it usually took seven months for parents to determine to take their child to receive thorough evaluations from the first visit to the child psychiatric clinic (66). Hence, to identify children with ASD in lower age groups, it is necessary to strengthen parents’ understanding of the relevant knowledge. Second, due to lacking of reliable biomarkers, the diagnosis of ASD is made primarily through interactive behavioral observations (16, 17). Unlike parent reports, comprehensive evaluations of children’s behavior by trained clinicians partly makes CHCTA more effective and efficient in identifying children at high likelihood of ASD. Third, rather than simply judge if a child has a specific type of behavior or not, CHCTA has adopted a multi-level scoring system that can precisely reflect children’s behavior deviation from normal development. Finally, the scale combines China’s national conditions and the behavioral performance characteristics of infants and young children, and designs activities that are more suitable for infants and young children, making the item contents of the scale more localized and having a higher social ecological validity.

However, the CHCTA tool also has several limitations need to be noted. Firstly, the assessment requires approximately 20 minutes to complete and must be administered and evaluated by trained professionals. Compared to parent-report measures, this process is relatively time-consuming and involves higher personnel training costs, which may limit its large-scale implementation. Nonetheless, professional and detailed observation can significantly reduce missed diagnoses caused by parental reporting bias. Secondly, although the total sample was randomly split into a scale development subset and a cut-off determination subset, this constitutes only internal cross-validation. Moreover, the subset used for cut-off determination was limited in size and characterized by an uneven age distribution, leaving the model susceptible to overfitting and optimistic bias. External validation would require ethical approval, data privacy safeguards, and inter-institutional data-sharing agreements—a time-intensive process infeasible within the present study timeline. Future research should validate the predictive performance of this cut-off in a fully independent, non-homologous, and larger sample. Thirdly, this scale demonstrates relatively limited specificity in balancing sensitivity and specificity. While it effectively identifies most children with ASD, its ability to distinguish ASD from DD remains suboptimal. To enhance diagnostic discrimination and mitigate false-positive and false-negative rates, future validation studies should integrate complementary multimodal assessment strategies—including parent interview questionnaires, expert clinical observations, and auxiliary diagnostic tools. Finally, this study did not employ internationally recognized gold-standard diagnostic tools such as ADOS-2 and ADI-R. Instead, the widely used CARS diagnostic scale in the Chinese mainland and comprehensive clinical diagnosis were adopted. While this improved instrument applicability and screening efficiency, it led to inconsistencies with other international studies, potentially limiting direct comparison of results. Additionally, for certain ASD groups, diagnostic outcomes around age 2 are characterized by instability and dynamic changes, which may constrain the external validity and generalizability of the findings.

## 5 Conclusions

This study presents preliminary psychometric evidence supporting the CHCTA as a promising early screening instrument for ASD in Chinese children. Grounded in an interactive, behavior-anchored assessment framework and incorporating a multi-tiered scoring system, the CHCTA demonstrates capacity to reliably identify children at elevated risk for ASD. Nevertheless, the optimal cut-off value derived from this study warrants further validation in external cohorts, and the specificity remains limited. Thus, the present conclusions should be considered exploratory in nature, and their interpretation and application require caution.

## Data Availability

The original contributions presented in the study are included in the article/supplementary material. Further inquiries can be directed to the corresponding author.
